# Actual use of PROMs in asthma and rhinitis recommended by guidelines in clinical settings: PROMUSE respiratory study

**DOI:** 10.3389/falgy.2026.1666241

**Published:** 2026-04-24

**Authors:** Ivan Cherrez-Ojeda, Jean Bousquet, Torsten Zuberbier, Juan C. Calderon, Pavel Kolkhir, Anastasiia Allenova, Andrey Allenov, Sergey Tkachenko, Florin Mihaltan, Ruxandra Ulmeanu, Mona Al-Ahmad, Mònica Rodriguez-González, Guillermo Wakida-Kusunoki, German D. Ramon, Maia Rukhadze, Désirée E. Larenas-Linnemann, Mitja Košnik, Juan Carlos Ivancevich, Luis Felipe Ensina, Nelson A. Rosario Filho, Violeta Kvedariene, Herberto Jose Chong-Neto, Ivan Tinoco, Anna Bedbrook, Gabriela Rodas-Valero, Marco Faytong-Haro, Marcus Maurer, Oliver Pfaar, Karla Robles-Velasco

**Affiliations:** 1 Institute of Allergology, Charité-Universitätsmedizin Berlin, Corporate Member of Freie Universität Berlin and Humboldt-Universität zu Berlin, Berlin, Germany; 2 Fraunhofer Institute for Translational Medicine and Pharmacology ITMP, Immunology and Allergology, Berlin, Germany; 3 Universidad Espíritu Santo, Samborondón, Ecuador; 4 Respiralab Research Group, Guayaquil, Ecuador; 5 Laboratory of Immune-Mediated Skin Diseases, Institute of Regenerative Medicine, Biomedical Science and Technology Park, First Moscow State Medical University Named After I.M. Sechenov (Sechenov University), Moscow, Russia; 6 Institute for Leadership and Health Management, I.M. Sechenov First Moscow State Medical University (Sechenov University), Moscow, Russia; 7 State Budgetary Healthcare Institution of the City of Moscow “City Polyclinic No. 210 of the Department of Health of the City of Moscow”, Moscow, Russia; 8 Federal State Budgetary Scientific Institution “N.A. Semashko National Research Institute of Public Health”, Moscow, Russia; 9 Russian Medical Academy of Сontinuous Professional Education of the Ministry of Health of the Russian Federation, Moscow, Russia; 10 National Institute of Pneumology M Nasta, Bucharest, Romania; 11 Integrated Pneumology and Bronchoscopy Center of Nord Pipera Hospital - Bucharest, Faculty of Medicine, Oradea, Romania; 12 Department of Microbiology, College of Medicine, University of Kuwait, Kuwait City, Kuwait; 13 Al-Rashed Allergy Center, Ministry of Health, Kuwait City, Kuwait; 14 Pediatric Allergy and Clinical Immunology, Hospital Español de México, Ciudad de México, Mexico; 15 Colegio Mexicano de Pediatras Especialistas en Inmunología Clínica y Alergia, Mexico City, México; 16 Hospital Italiano Regional del Sur, Bahía Blanca, Argentina; 17 Faculty of Medicine, Center Allergy & Immunology, Tbilisi, Georgia/Geomedi University, Tbilisi, Georgia; 18 Center of Excellence in Asthma and Allergy, Médica Sur Clinical Foundation and Hospital, Mexico City, Mexico; 19 University Clinic of Respiratory and Allergic Diseases Golnik, Golnik, Slovenia; 20 Servicio de Alergia e Immunologia Clinica Santa Isabel, Buenos Aires, Argentina; 21 Division of Allergy, Clinical Immunology and Rheumatology, Department of Pediatrics, Federal University of São Paulo, São Paulo, Brazil; 22 Urticaria Center of Reference and Excellence (UCARE), Department of Pediatrics, Federal University of Parana, Curitiba, Brazil; 23 Institute of Biomedical Sciences, Department of Pathology, Faculty of Medicine, Vilnius University, and Institute of Clinical Medicine, Clinic of Chest Diseases, Immunology and Allergology, Faculty of Medicine, Vilnius, Lithuania; 24 Department of Pediatrics, Hospital de Clínicas, Federal University of Paraná (UFPR), Curitiba, Brazil; 25 Centro de Alergia Tinoco, Machala, Ecuador; 26 MASK-air SAS, Montpellier, France; 27 Facultad de Investigación, Universidad Estatal de Milagro, Milagro, Guayas, Ecuador; 28 Instituto de Investigación, Universidad Agraria del Ecuador, Guayaquil, Ecuador; 29 Department of Otorhinolaryngology, Head and Neck Surgery, Section of Rhinology and Allergy, University Hospital Marburg, Philipps-Universität Marburg, Marburg, Germany

**Keywords:** allergic rhinitis, asthma, patient reported outcome measures, physicians, rhinosinusitis

## Abstract

**Rationale:**

Guidelines advise for the implementation of patient-reported outcomes (PROMs) to provide crucial insights into patients' perceptions of their disease burden, treatment needs, and quality of life. Despite their proven benefits in managing chronic respiratory diseases like asthma, allergic rhinitis (AR), and rhinosinusitis (RS), there is limited data on their adoption among physicians treating these conditions.

**Objectives:**

Our objective is to identify the utilization patterns of PROMs, together with the reasons for their usage and the barriers to their adoption among practitioners managing patients with asthma, AR, and RS.

**Methods:**

This was a cross-sectional observational study using a questionnaire encompassing all pertinent PROMs and disseminated to practitioners associated with the ARIA, UCARE, ADCARE, and ACARE networks. Individuals unfamiliar with PROMS or lacking prior experience with it were eliminated. Descriptive and analytical data were utilized, categorized by the frequency and type of PROMs applied. Stata 18.0 was utilized, with *p* < 0.05 indicating statistical significance.

**Results:**

A total of 439 practitioners participated, with PROMs predominantly utilized by physicians certified for over 30 years and by respiratory specialists (16.67% and 12.46%, respectively; *p* < 0.05). Pulmonologists exhibited the greatest utilization of asthma PROMs at 86%, while allergists predominantly employed AR and RS PROMs at 38.42% and 33.33%, respectively (*p* < 0.001). ACT (66.74%), RCAT (27.79%), and SNOTT22 (15.26%) were the predominant PROMs utilized primarily for asthma (79.19%), AR (51.23%), and RS (57.26%), respectively (*p* < 0.001). The foremost purposes for their application were disease control monitoring (93.39%) and evaluation of performance of therapy approaches (90.2%). The most significant barrier identified was time constraint, rated at 75.40% (*p* > 0.05 across all groups).

**Conclusions:**

The use of PROMs is suboptimal, primarily due to time limitations. It is imperative that methods be swiftly implemented to include these techniques into the therapeutic environment to attain enhanced outcomes.

## 1 Introduction

Recently, there has been an increasing emphasis on incorporating the patient's perspective in managing chronic conditions. One valuable tool for capturing this perspective is Patient-Reported Outcome Measurements (PROMs), which reflect patients' perceptions of the short- and long-term effects of their disease, well-being, medication needs, health-related quality of life (HRQoL), and satisfaction with treatment and outcomes (1). These reports come directly from the patients, without any influence or judgment from clinicians or other parties. The utility of PROMs has grown significantly in recent years and has proven beneficial in assessing conditions such as asthma, allergic rhinitis (AR), and chronic rhinosinusitis (CRS) (2).

Importantly, PROMs capture aspects of the patient's experience that may differ from the clinician's perception, as physicians often overestimate or underestimate the burden of symptoms in their patients' daily lives. For example, Panettieri et al. demonstrated that while 67% of patients with uncontrolled asthma were identified as such through the Asthma Control Test (ACT), physicians recognized uncontrolled asthma in only 44% of these cases (3). Similarly, in allergic rhinitis, Blaiss et al. reported discrepancies between healthcare providers and patients in identifying the most bothersome symptoms (4). Moreover, patients reported higher levels of productivity loss at work or school than healthcare providers estimated (4).

Asthma, AR, and CRS are chronic respiratory diseases with rising prevalence. For instance, the number of individuals with asthma increased from 226.9 million in 1990 to 262.41 million in 2019 (5). Approximately 20% of the global population is affected by AR (6), and 10% by CRS (7). These conditions not only impact health but also impose significant economic burdens due to absenteeism, presenteeism, and loss of productivity (8). In the most severe cases, these diseases can lead to complications and fatalities, particularly in children with asthma (9).

Implementing PROMs is critical for assessing the impact, level of control, and outcomes of management in these increasing patient populations (9–19). PROMs also help promptly identify patterns of poor response to treatment and facilitate data-driven decision-making (8). However, as reported in dermatological diseases, the adoption of PROMs remains limited; only 50% of physicians are aware of PROMs, and just 15% use them (2). Currently, there is limited information about the usage patterns and frequency of PROMs among physicians caring for patients with respiratory diseases. For this reason, the aim of this study is to determine the patterns of PROM use among practitioners managing patients with asthma, AR, and CRS, with the goal of identifying barriers, and further areas of learning for improving patient-centered care (9–21).

## 2 Methods

### 2.1 Questionnaire

The questionnaire was developed according to previously published guidelines (22). A steering committee for the PROMUSE project, composed of four experts and heads from four specialized allergy centers worldwide, reviewed the literature and developed the survey items, which integrated eight constructs to be assessed: demographics, knowledge about PROMs, frequency of use, PROM preferences, as well as satisfaction, physician training, attitudes, and barriers of using PROMs. This questionnaire consisted of fifty-three questions, which included multiple-choice questions, Likert and rating scales, and visual analogue scales. Although the questionnaire was developed through expert consensus and based on established literature, it has not undergone a formal psychometric validation process.

When asking about specific PROMs in asthma, we included the following tools: Asthma Quality of Life Questionnaire (AQLQ) (23), VAS (24), Asthma Control Test (ACT) (25), Mini-Asthma Quality of Life Questionnaire (mini-AQLQ) (26), ACQ (27), Asthma Symptoms Utility Index (ASUI) (28), AM/PM Asthma Symptoms Score (29), and Asthma Bother Profile (ABP) (30). When asking about allergic rhinitis and rhinosinusitis PROMs, we included these tools: RQLQ (31), VAS (32), EQ-5D (33), and Rhinitis Control Assessment Test (34).

### 2.2 Data collection

Considering the global importance of the use of PROMs to assess respiratory diseases, we sent the designed questionnaire to the heads of the following research networks: ARIA (Allergic Rhinitis and its Impact on Asthma), UCARE (Urticaria Centers of Reference and Excellence), ADCARE (Atopic Dermatitis Centers of Reference and Excellence), and ACARE (Angioedema Centers of Reference and Excellence).

The centers disseminated the survey to their physicians and to those of local and regional networks, encompassing not only respiratory clinics but also various healthcare facilities and professionals. Participants across these network centers and extended networks completed the survey. While this sampling strategy does not represent all the medical doctors or specialists in specific geographic areas, it is an expert sampling that collects information from worldwide medical care providers who commonly treat allergic diseases. Only practitioners managing patients with respiratory diseases were included.

The questionnaire was disseminated through different mailing servers and internal communication systems of the ARIA, UCARE, ADCARE, and ACARE networks. Due to the confidentiality of the member databases of these professional associations, the total number of physicians who received the invitation and the number who declined participation could not be determined. Therefore, response and refusal rates could not be calculated.

A purposive sampling strategy was used to reach physicians with recognized involvement in the management of asthma, allergic rhinitis, and rhinosinusitis within internationally established expert networks. Respondents were drawn from multiple continents, reflecting substantial geographic diversity. Europe accounted for the largest proportion of respondents (54.1%), followed by Latin America (27.7%) and the Middle East (13.8%). Smaller proportions were contributed by Asia (1.7%), North America (1.7%), Africa (0.9%), and Oceania (0.1%). No single country accounted for a majority of respondents, underscoring the global scope of the sample.

### 2.3 Ethics review

This study complies with the World Medical Association Declaration of Helsinki on Ethics. It was approved by the IRB “Comité de ética e Investigación en Seres Humanos (CEISH)” from Guayaquil-Ecuador (#HCK-CEISH-21-002). Informed consent was obtained from all participants before their voluntary participation in the survey. The participants could not be personally identified using the information collected in the study; thus, their anonymity was preserved, and their personal data were protected.

### 2.4 Analytical sample and statistical analysis

Health professionals who do not know PROMS or have never used them were excluded. Doctor-reported outcomes measured the use of PROMS. Data were collected between January and December 2023. Data were analysed for the overall group of health professionals and stratified by the frequency of use of PROMs, and the type of PROM used according to the respiratory disease treated.

Descriptive analyses were conducted to summarize participant characteristics, patterns of PROM use, perceived barriers, and training needs. All variables included in the analyses were categorical and are reported as frequencies and percentages according to the corresponding response categories.

Comparisons between groups, such as differences in the frequency of PROM use across provider characteristics and differences in the use of specific PROMs across disease areas, were assessed using chi-square tests of independence. These analyses were intended to identify associations between categorical variables and differences in distributions across groups, rather than to estimate effect sizes or draw population-level inferences. Analyses were performed using complete-case (observed) data, and no imputation for missing data was required. All statistical analyses were conducted using Stata version 18. Statistical significance was defined as a two-sided *p*-value < 0.05. Given the exploratory nature of the study and the use of a non-probabilistic purposive sampling strategy, results are primarily presented using *p*-values to indicate statistically significant associations, while effect sizes and confidence intervals were not emphasized.

## 3 Results

### 3.1 Patient demographics

A total of 439 respiratory health professionals reported the use of PROMs and completed the survey (32.40% of the sample). The demographic characteristics are shown in Table 1. There was no difference between categories of sex, age group, and type of consultation in terms of the frequency of PROM use. Specialists with 1–9 years post-board certification were less likely to consistently utilize PROMs (4.58%), whereas those with over 30 years post-certification were most likely to do so (16.67%) (*p* < 0.05). Furthermore, respiratory specialists always documented the utilization of PROMs (12.46%), in contrast to general physicians (0.0%) (*p* < 0.01). Overall, physicians indicated a more substantial utilization of PROMs (always) in AR (13.79%) and CRS (16.24%) compared to asthma (11.62%) (*p* < 0.01); however, there were no variations in the rates of Asthma PROMs usage (Supplementary Table S1).

**Table 1:** Demographic characteristics of participants included in the analysis and frequency of use of PROMs.

Demographic characteristics	Rarely	Sometimes	Often	Always	*p* value	Total (*n* = 439)
Sex					0.508	
Male	16 (8.60%)	76 (40.86%)	70 (37.63%)	24 (12.90%)		186
Female	29 (11.46%)	105 (41.50%)	96 (37.95%)	23 (9.09%)		253
Age Group					0.065	
20–29	2 (6.06%)	21 (63.64%)	10 (30.30%)	0 (0.00%)		33
30–39	13 (9.70%)	62 (46.27%)	50 (37.31%)	9 (6.72%)		134
40–49	13 (11.11%)	43 (36.75%)	43 (36.75%)	18 (15.39%)		117
50–59	8 (9.41%)	32 (37.65%)	37 (43.53%)	8 (9.41%)		85
60+	9 (12.86%)	23 (32.86%)	26 (37.14%)	12 (17.14%)		70
Type of consultation					0.801	
Public practice	14 (8.43%)	68 (40.96%)	66 (39.76%)	18 (10.84%)		166
Private practice	10 (11.25%)	35 (39.33%)	37 (41.57%)	7 (7.86%)		89
Both public and private	21 (11.41%)	78 (42.39%)	63 (34.24%)	22 (11.96%)		184
Years the provider has been a specialist					0.033	
1–9	8 (6.11%)	64 (48.85%)	53 (40.46%)	6 (4.58%)		131
10–19	15 (11.45%)	53 (40.46%)	48 (36.64%)	15 (11.45%)		131
20–29	9 (11.11%)	36 (44.44%)	26 (32.10%)	10 (12.35%)		81
30+	13 (13.54%)	28 (29.17%)	39 (40.62%)	16 (16.67%)		96
Specialty					0.006	
Yes, Specialty	39 (10.40%)	145 (38.67%)	144 (38.40%)	47 (12.53%)		375
No, General Physician	6 (9.37%)	36 (56.25%)	22 (34.37%)	0 (0.00%)		64
Respiratory Specialty (RS)					0.005	
Non-RS	13 (8.67%)	79 (52.67%)	47 (31.33%)	11 (7.33%)		150
Yes, RS	32 (11.07%)	102 (35.29%)	119 (41.18%)	36 (12.46%)		289

PROMs, Patient reported outcomes measurements.

### 3.2 Use of PROMs according to the “asthma” PROM users

Regarding specialists, Pulmonologists reported higher use of Asthma PROMs (86%) in comparison with Ear-Nose-Throat (ENT) specialists that reported the lowest use (1.89%). There was no statistical difference in the frequency of use (always 11.62% vs. rarely 9.19%) (Supplementary Table S1). Health professional users of asthma PROMs (Table 1) employed them significantly more often for monitoring disease control (95.13%), facilitating communication with patients (76.49%), and for research (61.35%) in contrast to non-users (*p* < 0.05). On the other hand, a higher proportion of physicians identified time constraints (77.03%) or lack of integration into clinical systems (60.81%) as main barriers to using them, but without statistical difference to non-users. Regarding areas interested in receiving further training/information, most areas were about how to choose which PROMs to use (81.08%) and how to interpret PROM scores (75.95%). There were no differences about how patients access and fill PROMs.

All specific Asthma PROM users reported different statistical patterns in the use of Asthma PROMs: ACT (Asthma Control Test) was the most used and AM/PM Asthma Symptoms Score the least used (79.19% and 10% respectively). Within this cohort, there were reports on the utilization of PROMs for allergic rhinitis and chronic rhinosinusitis; however, utilization was limited, with only the RCAT (23%) and SNOT22 (12%) demonstrating notable usage. A summary of the use of Asthma PROMs is shown in Tables 2, 3.

**Table 2:** Reasons to use, training needs, and barriers in implementing patient-reported outcome measurements in asthma, allergic rhinitis, and chronic rhinosinusitis.

Variable	Asthma	*p*-value	Allergic Rhinitis	*p*-value	Chronic Rhino-sinusitis	*p*-value	Overall
What do you use PROMs for?							
To facilitate decision making	316 (85.41%)	0.368	176 (86.70%)	0.289	96 (82.05%)	0.345	372 (84.74%)
To improve efficiency of consultation	296 (80.00%)	0.956	171 (84.24%)	0.038	90 (76.92%)	0.339	351 (79.95%)
To facilitate communication across different healthcare sectors	192 (51.89%)	0.535	111 (54.68%)	0.183	67 (57.26%)	0.129	225 (51.25%)
To facilitate communication with patients	283 (76.49%)	0.048	157 (77.34%)	0.241	86 (73.50%)	0.725	328 (74.71%)
To monitor disease control	352 (95.13%)	0.001	191 (94.09%)	0.587	108 (92.31%)	0.581	410 (93.39%)
To monitor disease severity	331 (89.46%)	0.195	175 (86.21%)	0.141	98 (83.76%)	0.054	389 (88.61%)
To monitor performance and therapeutic approach	336 (90.81%)	0.323	190 (93.60%)	0.027	105 (89.74%)	0.845	396 (90.20%)
For research	227 (61.35%)	0.047	143 (70.44%)	0.004	87 (74.36%)	0.004	278 (63.32%)
Other	26 (7.03%)	0.038	21 (10.35%)	0.129	12 (10.26%)	0.344	36 (8.20%)
For which of the following areas would you like to receive further training/information?							
What PROMS are	139 (37.57%)	0.249	84 (41.38%)	0.290	45 (38.46%)	0.946	170 (38.72%)
The benefits of using PROMS	217 (58.65%)	0.569	122 (60.10%)	0.730	68 (58.12%)	0.776	260 (59.22%)
The challenges of using PROMs	241 (65.13%)	0.653	138 (67.98%)	0.181	85 (72.65%)	0.035	284 (64.69%)
How to choose which PROMs to use	300 (81.08%)	0.274	171 (84.24%)	0.048	97 (82.91%)	0.388	352 (80.18%)
How to administer PROMs	232 (62.70%)	0.866	133 (65.52%)	0.287	75 (64.10%)	0.747	276 (62.87%)
How to calculate PROM scores	231 (62.43%)	0.833	124 (61.08%)	0.531	77 (65.81%)	0.408	275 (62.64%)
How to interpret PROM scores	281 (75.95%)	0.261	152 (74.88%)	0.976	88 (75.21%)	0.937	329 (74.94%)
Other further training areas	28 (7.57%)	0.338	19 (9.36%)	0.081	9 (7.69%)	0.756	31 (7.06%)
What are the main barriers to the use of PROMs?							
Time constraints	285 (77.03%)	0.067	154 (75.86%)	0.834	89 (76.07%)	0.844	331 (75.40%)
Mandated to complete	179 (48.38%)	0.214	110 (54.19%)	0.078	56 (47.86%)	0.650	218 (49.66%)
Sufficient understanding of the disease without PROMS	155 (41.89%)	0.514	88 (43.35%)	0.403	45 (38.46%)	0.477	181 (41.23%)
Patients dislike questionnaires	203 (54.87%)	0.975	118 (58.13%)	0.207	72 (61.54%)	0.092	241 (54.90%)
Uncertainty about reliability	144 (38.92%)	0.477	83 (40.89%)	0.619	50 (42.73%)	0.424	174 (39.64%)
Perceived as additional cost	99 (26.76%)	0.259	46 (22.66%)	0.171	29 (24.79%)	0.783	113 (25.74%)
Constrain doctor-patient relationship	69 (18.65%)	0.970	37 (18.23%)	0.822	24 (20.51%)	0.552	82 (18.68%)
Lack of integration into clinical systems	225 (60.81%)	0.074	127 (62.56%)	0.159	74 (63.25%)	0.275	259 (59.00%)
Lack of confidence in interpreting	122 (32.97%)	0.321	71 (34.97%)	0.671	42 (35.90%)	0.602	149 (33.94%)
Feel uncomfortable	112 (30.27%)	0.831	64 (31.53%)	0.536	40 (34.19%)	0.257	132 (30.07%)
Not available in the native language of my patients	157 (42.43%)	0.610	85 (41.87%)	0.987	50 (42.73%)	0.833	184 (41.91%)
Not available for certain groups	179 (48.38%)	0.598	106 (52.22%)	0.088	54 (46.15%)	0.671	210 (47.84%)
Not suitable for obtaining the information I need	102 (27.67%)	0.996	68 (33.50%)	0.010	40 (34.19%)	0.061	121 (27.56%)
Too complicated to fill in	120 (32.43%)	0.929	64 (31.53%)	0.734	44 (37.61%)	0.156	142 (32.35%)
Too complicated to evaluate/score	105 (28.38%)	0.887	57 (28.08%)	0.942	33 (28.20%)	0.991	124 (28.25%)
How patients access PROMs							
Clinical Systems	128 (34.59%)	0.254	73 (35.96%)	0.308	45 (38.46%)	0.183	147 (33.48%)
Online	240 (64.87%)	0.121	136 (66.99%)	0.139	71 (60.68%)	0.489	278 (63.33%)
Paper	286 (77.30%)	0.260	150 (73.89%)	0.269	95 (91.20%)	0.147	335 (76.31%)
Other	19 (5.13%)	0.821	15 (7.39%)	0.061	8 (6.84%)	0.365	23 (5.24%)
How patients fill forms							
By paper	312 (84.32%)	0.342	167 (82.27%)	0.484	101 (86.32%)	0.353	367 (83.60%)
Electronically	161 (43.51%)	0.828	98 (48.28%)	0.075	47 (40.17%)	0.364	192 (43.74%)

**Table 3:** Frequency of use of specific PROMs across asthma, allergic rhinitis, and chronic rhinosinusitis.

Specific Asthma PROM that provider employs	Asthma	*p*-value	Allergic Rhinitis	*p*-value	Chronic Rhino-sinusitis	*p*-value	Overall
AQLQ	155 (41.89%)	0.000	58 (28.57%)	0.006	32 (27.35%)	0.035	155 (35.31%)
VAS Asthma	69 (18.65%)	0.000	39 (19.21%)	0.062	20 (17.09%)	0.633	69 (15.72%)
ACT Asthma	293 (79.19%)	0.000	111 (54.68%)	0.000	65 (55.56%)	0.003	293 (66.74%)
Mini AQLQ	97 (26.22%)	0.000	42 (20.69%)	0.510	25 (21.37%)	0.825	97 (22.10%)
ACQ	99 (26.76%)	0.000	36 (17.73%)	0.025	22 (18.80%)	0.257	99 (22.55%)
ASUI	36 (9.73%)	0.007	14 (6.90%)	0.356	11 (9.40%)	0.580	36 (8.20%)
AM PM Asthma Symptom Score	37 (10.00%)	0.006	14 (6.90%)	0.284	7 (5.98%)	0.266	37 (8.43%)
ABP Asthma Bother Profile	26 (7.03%)	0.023	14 (6.90%)	0.423	10 (8.55%)	0.160	26 (5.93%)
Other Asthma PROM	20 (5.40%)	0.048	10 (4.93%)	0.730	4 (3.42%)	0.491	20 (4.56%)
Specific Allergic Rhinitis PROM							
RQLQ	56 (15.13%)	0.000	70 (34.48%)	0.000	41 (35.04%)	0.000	83 (18.91%)
VAS	75 (20.27%)	0.000	90 (44.33%)	0.000	64 (54.70%)	0.000	108 (24.60%)
EQ5D	26 (7.03%)	0.038	31 (15.27%)	0.000	15 (12.82%)	0.033	36 (8.20%)
RCAT	86 (23.24%)	0.000	104 (51.23%)	0.000	59 (50.43%)	0.000	122 (27.79%)
Other Allergic Rhinitis PROM	27 (7.30%)	0.000	33 (16.26%)	0.000	20 (17.09%)	0.001	42 (9.57%)
Specific Rhinosinusitis PROM							
SNOT22	45 (12.16%)	0.000	51 (25.12%)	0.000	67 (57.26%)	0.000	67 (15.26%)
SNOT16 ARS	15 (4.05%)	0.243	16 (7.88%)	0.002	20 (17.09%)	0.000	20 (4.56%)
VAS Rhinosinusitis	38 (10.27%)	0.000	44 (21.67%)	0.000	62 (52.99%)	0.000	62 (14.12%)
Other Rhinosinusitis PROM	5 (1.35%)	0.346	3 (1.48%)	0.856	7 (5.98%)	0.000	7 (1.59%)

AQLQ, asthma quality of life questionnaire; VAS, visual analogue scale; ACT, asthma control test; mini-AQLQ, mini-asthma quality of life questionnaire; ACQ, asthma control questionnaire; ASUI, asthma symptoms utility index; AM/PM (morning/evening) Asthma Symptom Score; ABP, asthma bother profile; EQ-5D, EuroQol- 5 Dimension; RCAT, rhinitis control assessment test; RQLQ, rhinoconjunctivitis quality of life questionnaire; SNOT16 ARS, Sino-Nasal Outcome Test 16; SNOT22, Sino-nasal Outcome Test-22.

### 3.3 Use of PROMs according to the “allergic rhinitis” users

Most users of Allergic Rhinitis PROMs were allergists (38.42%), and around 14% of these users always used them compared with 5.42% who rarely used them (*p* < 0.01, Supplementary Table S1). Most Allergic Rhinitis PROM users employed them (i) to improve the efficiency of consultation, (ii) to monitor performance and therapeutic approach and (iii) for research (84.24, 93.60%, and 70.44% respectively), (*p* < 0.05, Table 2). A higher proportion of Allergic Rhinitis PROM users would like to receive more training/information on how to choose them (84.24%) and how to interpret them (74.88%) (*p* > 0.05).

“*Not suitable for obtaining the information needed*” was the only barrier with a statistical difference (33.50%, *p* = 0.01). As is the case of Asthma PROM users, there was no statistical difference in how patients accessed and filled in Allergic Rhinitis PROMs.

All Allergic Rhinitis PROM users reported statistically different trends in the use of Allergic Rhinitis PROMs: the most frequently used was RCAT and the least used EQ5D (51.23% and 15.27% respectively, *p* < 0.01).

Conversely, ACT was the most utilized PROM among this group of physicians (54.86%), but AQLQ (28.57%) and ACQ were the least utilized (28.57% and 17.73%, *p* < 0.05). Ultimately, SNOT22, SNOT16ARS, and VAS Rhinosinusitis were utilized by approximately 25% less of these clinicians (*p* < 0.01), see more details in Tables 2, 3.

### 3.3 Use of PROMs according to the “rhinosinusitis” users

Most users of Rhinosinusitis PROMs were respiratory specialists, predominantly allergists (33%), who frequently utilized these PROMs in over 50% of patients (*p* < 0.05 Supplementary Table S1). SNOT22 and VAS for rhinosinusitis were the most often utilized patient-reported outcome measures, at 57% and 53% respectively. The primary significant applications of these PROMs were for research (74.36%, *p* < 0.05) and for assessing disease severity (84%, *p* 0.054). Additionally, clinicians expressed a desire for enhanced training and information regarding “the challenges of utilizing PROMs” (72.65%). A greater percentage of physicians recognized primary obstacles to utilizing PROMs, including patient dislike to questionnaires (61.54%) and time limitations (76.07%). Nonetheless, no statistical differences were seen as compared to non-users (Table 2). Also, there was no difference between access for patients or filling in forms; this was similar to Asthma and Allergic Rhinitis PROM users (Table 2).

All specific Allergic Rhinitis PROMs showed a different pattern between users and non-users, specifically VAS which was the most used (55%) followed by RCAT (50%). Regarding Asthma PROMs, ACT was highly used (56%) (*P* < 0.05).

## 4 Discussion

Currently, there is increasing interest in the use of Patient Reported Outcomes Measurements (PROMs) in the respiratory field, beyond their original role in clinical trials. Our study found that nearly 48.52% of physicians frequently use PROMs for Asthma, AR, and RS, which contrasts with other clinical areas. For example, PROM use in oncology is reported in fewer than 28% of physicians (35), while in immunology, only 15% of clinicians use PROMs for conditions such as atopic dermatitis or chronic urticaria (36). In orthopedics, approximately 35%–46% of surgeons actively collect PROMs in routine practice (37). Among general practitioners, one survey found that 77% use at least one PROM (38). Given the complexity of respiratory management, these results underscore a pivotal shift toward using PROMs to enhance the understanding of patient needs and optimize longitudinal care (35).

PROMs (self-reported measures) enable patients to capture their disease perceptions, and they also emphasize patient-centered care, focusing on what matters to the post-intervention phase of patients, a viewpoint often overlooked by physicians (39). These tools are increasingly utilized in respiratory disease management and research for their valuable insights into patient health and treatment perspectives. In asthma, integrating patient-centered outcomes could resolve discrepancies by addressing patient knowledge, individualized treatment plans, and simpler regimens (40). For AR, its non-threatening nature leads to patients not seeking medical advice, and to physicians underestimating the disease's disruptive impact, which varies with symptom severity (41, 42). There are significant differences between patient and physician expectations in allergic rhinitis management, underscoring the need for improved care (42).

Using PROMs in asthma clinical practice offers several benefits. Physicians often overestimate patients' asthma control compared to objective measures (43). Validated PROMs like the Asthma Control Questionnaire (ACQ) and Asthma Control Test (ACT) help identify patients needing treatment adjustments. An ACQ includes seven questions on symptoms, predicted FEV1%, and rescue bronchodilator use. However, our findings indicate that ACT and AQLQ are the most employed, likely because AQLQ does not require FEV1 measurements (44). Although there are no studies specifically assessing patients' preferences regarding asthma PROMs, previous research provides valuable insights into patient perspectives, particularly in severe asthma. Qualitative studies show that patients with severe asthma experience a substantial disease burden beyond symptom control, encompassing emotional, social, financial, and intimacy-related aspects of quality of life, which are often insufficiently captured by commonly used PROMs such as the ACT or ACQ (45). Sexual and intimacy-related quality of life, in particular, remains underrepresented in existing asthma PROMs (45). In addition, discrepancies between patient-perceived asthma control and objective disease indicators have been consistently reported, with many patients describing their asthma as “well controlled” despite frequent symptoms or rescue medication use (46). This suggests that numerical scores alone may not fully reflect patients' lived experience or perceived risk. Furthermore, qualitative evidence supports a shift from a predominantly medical-centered use of PROMs toward more holistic, patient-engaging approaches, including digital integration and incorporation into patient education (47). Taken together, these findings suggest that while current PROMs are valuable for clinical decision-making, patients may benefit from more comprehensive and patient-centered tools. Interestingly, we found a trend of PROM usage associated with the practitioner's age.

A systematic review identified the Rhinitis Control Assessment Test (RCAT) and the Control of Allergic Rhinitis and Asthma Test (CARAT) as the most validated disease control strategies. The VAS scale is also widely used due to its simplicity. In our study, RCAT was the most used PROM for rhinitis, while CARAT was excluded given that it assesses both asthma and allergic rhinitis rather than each condition separately. Using CARAT as a composite PROM may potentially reduce assessment time, addressing a primary constraint reported by our participants. The RQLQ and mini-RQLQ have been extensively validated in AR, largely due to the pioneering work of their developers (48). In our study, this PROM was used by about one-third of respondents. A recent review highlighted the predictive capabilities of SNOT-22 as well as its utility for clinicians in diagnosis, workup, and management. In our study, this tool was the most utilized by physicians. However, most predictions are supported by low-evidence studies, and SNOT-22 is yet to be validated as a predictive tool (49, 50).

Our findings suggest a correlation between practitioner age and PROM usage. Specifically, the age-related patterns observed appear more pronounced among respiratory specialists than non-specialists. Louis et al. found no statistically significant influence of healthcare practitioners' seniority on PROM use (51). Younger physicians are more inclined to adopt patient-centered approaches like PROMs and may embrace new technologies and innovations in health care, given their training emphasis on patient-centered care and exposure to research supporting PROM value in clinical settings (52, 53).

Our analysis revealed that the age gradient in the use of PROMs for respiratory diseases is primarily driven by respiratory specialists (allergists, pulmonologists, and ENT specialists). *post-hoc* analyses showed that respiratory specialists were more likely to use PROMs for allergic respiratory diseases regardless of age. For example, PROM use among respiratory specialists increased from 7.69% in the youngest age group (27–35, 39) to 29.33% in the middle-aged group (53–61). In contrast, non-respiratory specialists showed a modest decline in PROM usage with increasing age, indicating a less pronounced age-related trend. This pattern may reflect differences in training and professional focus: older respiratory specialists value PROMs more, due to increased training, while younger non-respiratory specialists may place greater value on PROMs due to accumulated clinical experience and disease-specific training, whereas younger non-respiratory specialists may be more familiar with PROMs as a result of recent curricular exposure. Since respiratory specialists appear to drive the overall age gradient in PROM use, and older specialists report higher PROM adoption, further investigation of additional contributing factors, such as clinical workload, patient complexity, and institutional practices, is warranted. Previous studies have shown that older physicians (>60%) demonstrate higher levels of empathy and trust, with nearly half of patients reported satisfaction with the physician-patient relationship (*p* < 0.05) (54). However, large age gaps between physicians and patients may still hinder effective communication (55). Taken together, this “divergence by specialty” suggests that specialized training and long-term experience in respiratory care may outweigh the potential advantage of more recent PROM-focused curricula among younger generalists. Moreover, female practitioners reported greater use of PROMs in asthma; this aligns with literature suggesting a tendency toward prioritizing patient-centered outcomes and less dominant communication styles in this demographic (47, 56, 57).

A significant clinical discordance exists in how patients and physicians assess disease impact. Our findings align with Meltzer et al., who observed that physicians tend to underestimate the prevalence of ocular symptoms compared to patients (37% vs. 57%) while overestimating the impact of nasal congestion (86% vs. 67%) (58). This discrepancy often is associated with a clinical preference for treating nasal symptoms over the symptoms patients find most bothersome. Despite evidence that AR symptoms significantly impair HRQoL, it is frequently viewed as a lower priority by clinicians, potentially leading to an incomplete understanding of its true burden (58). Because conventional clinical indices often correlate poorly with the lived experience of AR, researchers suggest that HRQoL should be measured directly. However, a notable gap remains in practice: while systematic reviews recommend the Rhinitis Quality of Life Questionnaire (RQLQ) as a primary tool, only of our study participants reported utilizing it (54).

Ta et al. (62) found no correlation between objective tests measuring physiological parameters and treatment effectiveness and patients' perceptions of their sinus-nasal disorder. This disparity limits the use of PROMs. However, a longitudinal use of PROMs can enhance patients' quality of life, improve patient-physician communication, reduce emergency visits, and support shared decision-making, as clinicians often underestimate patients' symptoms and their impact (59), which frequently go unnoticed (60). It is crucial to recognize that patients better understand the daily impact of AR, and physicians need to educate patients about the potential effects of AR on vision and daily activities. Additionally, physicians should pay more attention to patients' complaints and adopt a holistic approach to disease management (61).

Our study identified and confirmed significant barriers to PROM use, including time constraints, lack of integration into clinical systems, and the perception that patients dislike questionnaires. While interest in training is high (≈70%), the technical burden of administration and interpretation remains a significant hurdle for nearly a third of respondents. This underscores the need for automated scoring systems or digital integration. Louis et al. (47) found similar results about training to use PROMs (80%) as one of the top 3 suggestions to improve the routine use of PROMs.

With the recent technological development, one option could be the use of electronic questionnaires, being quicker and more convenient for physicians and patients to fill out. Also, some features can be used, e.g., restrictions on the answers to meet relevant criteria, avoiding missing values, automatic scoring, and intuitive assessments (63). In other areas, such as breast cancer, the time and information collected via electronic devices were the same when compared to paper-based methods. There were similar rates of use in women with low health literacy (64), and the possibility of integrating health information technology for collecting PROMs (35). In addition to countering time constraints, real-time digital tools can be used by patients prior to their appointments.

The MASK-air app for rhinitis and asthma allows patients to monitor symptoms, track medication use, and receive personalized feedback, while also facilitating communication with healthcare providers to improve care coordination (65–67). These technologies enhance chronic care by providing public information, monitoring and treating diseases, as well as supporting patient carers (68). Although 60% of surveyed individuals believe patients dislike using PROMs, there is a perception gap between physicians and patients regarding their longitudinal use. Abernathy et al. found that 88% of patients were satisfied with using a longitudinal e/Tablet data-collection system for symptoms and quality of life assessment, with 74% stating it helped them recall symptoms to report (59). Another study by Fida et al. showed that patients with atopic dermatitis had positive perceptions toward PROMs, and PROMs were simple to understand, were relevant to their illness, and facilitated communication with their doctors (69).

PROMs and quality of life (QoL) data may strengthen clinical practice by pinpointing issues, promoting collaborative decision-making, and enhancing patient satisfaction. This data collection strategy is applicable to telehealth and other remote healthcare services. Notwithstanding implementation obstacles, present-day examples can inform doctors (70, 71). Additional study is required to enhance comprehension of patient knowledge, attitudes, perceptions, experiences, and satisfaction about PROMs in respiratory medical field, as well as to ascertain the primary determinants of perceived barriers among clinicians. The findings, together with medical education on the advantages of PROMs and addressing the primary perceived hurdles, should alleviate the notion that patients oppose their utilization and the barriers perceived by physicians.

This study has several limitations. Because the survey was distributed through confidential mailing systems of international professional associations, response and refusal rates could not be estimated, which may introduce selection bias toward physicians with greater engagement in PROM-related activities. In addition, physicians affiliated with international networks such as ARIA, UCARE, ADCARE, and ACARE may be more research-oriented and more familiar with guideline-based care than the average healthcare provider, which may further limit the extrapolation of the findings to routine clinical practice.

Although the survey was disseminated through angioedema, atopic dermatitis, and urticaria networks (UCARE, ADCARE, and ACARE), approximately one-third of respondents (32.40%) were respiratory specialists involved in the care of patients with asthma, allergic rhinitis, and rhinosinusitis (Table 1). The results may therefore not represent all respiratory practices, particularly in less specialized or non-research-oriented healthcare settings. Physicians from specialized centers treating asthma, rhinitis, and rhinosinusitis are likely to use PROMs more frequently than primary care physicians or non-specialists, which may lead to an overestimation of PROM usage in routine clinical practice.

The inclusion of respondents from countries and continents with heterogeneous healthcare systems may introduce contextual variability related to differences in healthcare organization, access to resources, and integration of PROMs into routine practice. Because the study was designed to describe overall global patterns rather than to compare healthcare systems, and because respondent numbers were unevenly distributed across regions, country- or continent-specific analyses were not performed. These differences should be considered when interpreting the findings. In addition, the exploratory nature of the study and the use of a non-probabilistic purposive sampling strategy do not allow population-level inference, and the findings should be interpreted as indicative of patterns rather than representative estimates.

Furthermore, the questionnaire was not formally validated. A key strength of this study is that even among specialized and guideline-oriented centers, PROM usage remains suboptimal, underscoring the need to improve physician familiarity, training, and implementation strategies for PROMs in clinical care.

With these limitations in mind, this study represents one of the first international efforts to describe patterns of PROM use, perceived barriers, and training needs across multiple respiratory conditions within specialized clinical networks. The findings are intended to serve as a starting point for future research using different methodological approaches, including probabilistic sampling, longitudinal designs, qualitative studies, and implementation-focused evaluations, to further assess PROM adoption in diverse healthcare settings.

## 5 Conclusion

In clinical practice, PROMs have been demonstrated to improve treatment outcomes, management, and prognosis for patients with respiratory diseases. The results of our study show that their utilization remains suboptimal due to the challenges associated with integrating existing workflows, the complexity of the PROMs, time constraints, and a lack of training. PROMs must be implemented at a high level in routine clinical Respiratory care. We acknowledge that more physician education and information about these PROMs are urgently needed.

## Data Availability

The datasets presented in this study can be found in online repositories. The names of the repository/repositories and accession number(s) can be found in the article/Supplementary Material.
