# Supplementary material for: Actual use of PROMs in asthma and rhinitis recommended by guidelines in clinical settings: PROMUSE respiratory study
Source: Front Allergy. 2026 Apr 24;7:1666241. doi: 10.3389/falgy.2026.1666241 (PMC13154597; doi:10.3389/falgy.2026.1666241)
Supplement: Supplementary file 1 [file Table1.docx]

**Table S1.** Distribution of Patient-Reported Outcome Measure (PROM) usage across specialty type and asthma, allergic rhinitis, and chronic rhinosinusitis conditions.

| Variable | Asthma  (n=370) | p-value | Allergic rhinitis  (n=203) | p-value | Chronic Rhinosinusitis  (n=117) | p-value | Overall (n=439) |
| --- | --- | --- | --- | --- | --- | --- | --- |
| Non-Respiratory Specialty | 120 (32.43%) | 0.000 | 72 (35.47%) | 0.000 | 36 (30.77%) | 0.000 | 150 (34.17%) |
| Respiratory Specialty |  |  |  |  |  |  |  |
| ENT | 7 (1.89%) |  | 20 (9.85%) |  | 23 (19.66%) |  | 28 (6.38%) |
| Allergist | 137 (70.00%) |  | 78 (38.42%) |  | 39 (33.33%) |  | 153 (34.85%) |
| Pulmonologist | 106 (86.00%) |  | 33 (16.26%) |  | 19 (16.24%) |  | 108 (24.60%) |
| Any PROM Use (Frequency) |  | 0.091 |  | 0.001 |  | 0.000 |  |
| Rarely | 34 (9.19%) |  | 11 (5.42%) |  | 8 (6.84%) |  | 45 (10.25%) |
| Sometimes | 148 (40.00%) |  | 75 (36.95%) |  | 31 (26.50%) |  | 181 (41.23%) |
| Often | 145 (39.19%) |  | 89 (43.84%) |  | 59 (50.43%) |  | 166 (37.81%) |
| Always | 43 (11.62%) |  | 28 (13.79%) |  | 19 (16.24%) |  | 47 (10.71%) |
